# Prognostic Factors of Mortality among Adult Patients on Antiretroviral Therapy in India: A Hospital Based Retrospective Cohort Study

**DOI:** 10.1155/2019/1419604

**Published:** 2019-01-21

**Authors:** Nitin Joseph, Ushasti Sinha, Nishtha Tiwari, Pritha Ghosh, Patneedi Sindhu

**Affiliations:** ^1^Department of Community Medicine, Kasturba Medical College, Light House Hill Road, Manipal Academy of Higher Education, Mangalore, India; ^2^Kasturba Medical College, Light House Hill Road, Manipal Academy of Higher Education, Mangalore, India

## Abstract

**Introduction:**

HIV related deaths still continue to occur in large numbers in spite of good quality drugs being freely available in India. This study was therefore done to assess the prognostic factors of mortality among people living with HIV (PLHIV) on antiretroviral therapy (ART). This would help in planning strategies for further improving their survival.

**Materials and Methods:**

Record based data from baseline and follow-up visits of a cohort of patients aged above 14 years on ART was retrospectively reviewed over a seven-year period. The Kaplan-Meier models were used to estimate life time survival probability, and Cox proportional hazard regression model was used to determine independent prognostic factors of death, among patients, after initiation of ART.

**Results:**

Mean age of the total 285 patients enrolled in this study was 45.8±9.7 years. Mean duration of treatment on ART was 1127±611.8 days. During the follow-up period, 44/285(15.4%) patients died, resulting in incidence density of death rate as 3.12 per 100 person years. Good adherence with treatment was reported by 267(93.7%) patients. Nearly half of the deaths, i.e., 21(47.7%), occurred within three months of them starting ART. The mean survival time after initiation of ART was 2084.0±55.3 days (95% CI is 1975.5-2192.5). The presence of opportunistic infections (OIs) and tuberculosis before and poor/average adherence to ART and alcohol usage after starting ART were independent prognostic factors of mortality among patients.

**Conclusion:**

Several prognostic factors influencing mortality among adult HIV patients receiving treatment were identified in this study. Screening efforts is essential in early detection and management of OIs among PLHIV. Good counselling and monitoring is recommended to improve adherence and also to prevent alcohol usage after initiation of ART. Such measures would help in further reducing mortality among HIV patients in the settings.

## 1. Introduction

India stands third with respect to HIV epidemic in the world. About 2.1 million people are estimated to be living with HIV in India. The number of deaths due to AIDS related illnesses in India was estimated to be around 62,000 people in the year 2016 [1].

Free antiretroviral therapy (ART) is being provided by National AIDS Control Organization (NACO) of India since 2004. With the introduction of Highly Active Antiretroviral Therapy (HAART), there has been a sizable reduction of HIV associated mortality in developing countries [2, 3]. In India, a 54% decline in AIDS related deaths was reported between 2007 and 2015 [4]. However, reasons for the occurrence of so many deaths in 2016 require investigation.

Mortality at an early stage has been reported among patients soon after initiation of ART which is unlikely to be related to drug efficacy [5–7]. Hence there are several other factors which indirectly contribute to mortality among PLHIV (people living with HIV) which are often poorly understood [8]. Factors such as poor therapy adherence, drug toxicity, and presence of substance abuse and addiction, limit the success of HAART and affect the quality of self-care among PLHIV [9].

Identification and rectification of these modifiable factors during routine clinical care might help in prolonging survival thereby preventing more deaths among PLHIV. This study therefore aims to identify prognostic factors influencing mortality among a cohort of HIV infected adult patients on ART.

## 2. Materials and Methods

This retrospective cohort study was conducted in May 2017 in a major tertiary care hospital affiliated to a medical college in the city of Mangalore of Karnataka state in southern India. The approval to do this study was obtained from the Institutional Ethics Committee. Then, the permission to do the study at the setting was taken from the medical superintendent of the respective hospital.

The secondary data of all confirmed patients of HIV started on ART over a seven year follow-up period from January 2010 till December 2016 was collected by the investigators. The information regarding sociodemographic details of the patients, age at diagnosis of HIV, age at starting ART, functional status of patients, substance usage, associated OIs, WHO clinical staging of the patient, haematological reports, height, weight of the patient, and health status of the spouse were noted down in a predesigned content validated pro forma. The number of follow-up visits to the treatment centre, type of ART regimen, adherence to treatment, chemoprophylaxis regimen, and outcome of management whether survived or died was also recorded. No personal identifier was stated on the data collection form.

The cohort constituted patients aged above 14 years and who underwent at least two visits to the treatment centre.

Transferred out patients and lost to follow-up patients whose outcome following treatment was not known were censored. Patients who did not return for follow-up visits for more than three consecutive months were considered as lost to follow-up [10].

Patients who had already started treatment with ART before the period of observation of this study and those aged below 15 years were excluded.

The most recent laboratory results before starting ART was used as a base line value. In patients where this information was unavailable, the results obtained within a month of ART initiation were recorded as baseline values [11].

Body mass Index (BMI) was categorized as per Asian classification. Adherence to ART more than 95% was considered good, between 85 and 94% as average and less than 85% as poor adherence with treatment [11].

The survival time was calculated in days between the date of treatment initiation and the date of the event (death) or date of censoring [12]. Data were censored on 26^th^ May 2017.

Data were entered and analyzed using* * SPSS Inc., Chicago, IL, USA, version 16.0.

Descriptive statistics were used to describe the characteristics of the cohort. Person years of observation (PYO) and incidence rates were also calculated for various periods. The Kaplan-Meier curves were generated to estimate life time survival probability of patients.

Univariate analysis was done using Chi square test, unpaired t test, and Analysis of Variance. All the risk factors significant at 0.15 level qualified entry in the multivariable model and were excluded using a backward stepwise elimination procedure to identify the true predictors. Cox proportional hazard regression model was used to calculate the adjusted hazard rate and to determine independent prognostic factors of mortality among patients after initiation of ART. p ≤ 0.05 was considered statistically significant association.

## 3. Results

Mean age of the 285 patients enrolled in this study was 45.8±9.7 years with age ranging from 18 years to 76 years (Table 1). Mode of transmission of HIV in most of the patients was by hetero sexual contact 278(97.5%). The other modes of transmission were by intravenous drug usage in 3, blood transfusion in 2, and homo sexual contact and from mother to child transmission in one case each. Type of virus involved was HIV 1 in most patients 282(98.9%) and HIV 2 among the remaining 3 (1.1%) patients.

Median BMI (Kg per sq.mtrs) of patients was 20.1(IQR 17.7-22.9) before the start of ART (n=257), 20.7(IQR 18.4-23.6) after 6 months (n=249), 21.2(IQR 18.6-23.7) after 12 months (n=242), 21.1(IQR 18.6-23.1) after 24 months (n=178), 21.3(IQR 18.2-23.5) after 36 months (n=124), 21.4(IQR 18.3-23.2) after 48 months (n=105), and 21.5(IQR 18.2-23.6) after 60 months (n=67) of starting ART.

Around 3% and 7% increase of BMI from baseline median level was observed after 6 months and 60 months of ART, respectively.

Median CD4 count (cells per cu.mm) of patients was 364(IQR 201-498.5) before the start of ART (n=285), 410(IQR 314-574.5) after 6 months (n=254), 494(IQR 355-656.5) after 12 months (n=241), 562(IQR 401-728) after 24 months (n=175), 584.5(IQR 432-770) after 36 months (n=130), 575.5(IQR 418-754.5) after 48 months (n=106), and 563.5(IQR 450-722.5) after 60 months (n=62) of starting ART. The CD4 count before the start of ART was <200 cells per cu.mm of blood in 71(24.9%) patients.

13.6% and 59.4% increase of CD4 count from baseline median level were observed after 6 months and 48 months of ART, respectively.

The median CD4 count of patients (cells per cu.mm) at the time of death (n=44) was 210(IQR 128-375.5).

During the follow-up period, CD4 count remained the same or was less than the baseline values among 67(23.5%) patients, yielding an immunologic treatment failure rate of 41.5/100 person years of observation (PYO).

Death was reported among 6(85.7%) patients with CD4 count less than 100 cells per cu.mm of blood in comparison to 38(13.7%) patients with CD4 count ≥100 cells per cu.mm of blood (X^2^=27.1, p<0.001).

Mean haemoglobin values (gms per dl) before start of ART were 12.1±2.4 among patients.

Mean age of patients at the start of ART was 42.9±9.6 years (Table 1). Mean duration of treatment on first line drugs was 396.5±174.3 days.

Good adherence with ART was seen among 238(95.2%) patients without any side effects with ART compared to 29(82.9%) among those with side effects (X^2^=7.9, p=0.005).

Extra-pulmonary and pulmonary tuberculosis were the most common OI before and after starting ART, respectively (Table 2).

Cause of death was known in 13 patients. It is comprised of cardiac causes in 3, pneumonia in 6, and tuberculosis, and encephalitis in 2 patients each.

Majority of deaths, i.e., 21(47.7%), occurred within three months of starting ART. The proportion of patients who died was 7.4%, 1.1%, 1.1%, 1.9%, 2.4%, and 2.4% at <3 months, 3-6 months, 6 months to 1 year, 1-2 years, 2-3 years, and ≥3 years of treatment with ART, respectively. In terms of person years it was 3.2, 0.4, 0.2, 0.1, 0.1, and 0.1, respectively, during these periods of treatment with ART.

During the follow-up period, a total of 44/285(15.4%) patients died. The proportion of patients who died was 4.9%, 1.5%, 1.1%, 2.3%, 1.2%, 0%, and 5.5% at <3 months, 3-6 months, 6 months to 1 year, 1-2 years, 2-3 years, 3-4 years, and ≥4 years, respectively, following the diagnosis.

The death rate was 44 deaths/880.79 person years of treatment X 100 = 4.99 per 100 person years of treatment with ART. The death rate was 44/1407.8 person years since diagnosis X 100 = 3.12 per 100 person years.

The time between diagnosis of HIV and death was ≤100 days in 14(31.8%), 101 days to 1 year 8(18.2%), 1 year to 5 years in 12(27.3%), and more than 5 years in 10(22.7%) patients.

Out of the 44 deaths reported, the most recent CD4 count (cells per cu.mm) before death was <100, 100-199, 200-299, 300-399, 400-499, and ≥500 in 6(13.6%), 15(34.1%), 8(18.2%), 6(13.6%), 3(6.9%), and 6(13.6%), respectively.

The median duration of taking ART among the patients who died (n=44) was 110 (IQR 25.2-780) days.

The mean duration of taking ART (days) was 36.5, 335.3, 392.9, 490.2, 550, and 1207.5 among patients whose most recent CD4 count (cells per cu.mm) before death was <100, 100-199, 200-299, 300-399, 400-499, and ≥500, respectively (F=3.345, p=0.013).

Therefore patients who died with a low CD4 count were on ART for a much shorter time than those who died with a higher CD4 count. This implies that deaths at low CD4 count were caused by patients starting ART, too late to benefit.

The median survival time among the patients who died (n=44) was 380.5 (IQR 44-1756.2) days.

The mean survival time (days) following diagnosis with HIV was 401, 584.7, 1235.2, 757.8, 2719, and 1606.2 among patients whose most recent CD4 count (cells per cu.mm) before death was <100, 100-199, 200-299, 300-399, 400-499, and ≥500, respectively (F=2.494, p=0.048).

The mean survival time (days) following diagnosis with HIV was 1401±1393.8 days (or 46.7 months) and median survival time was 790 days (IQR 148-2667) for patients whose most recent CD4 count before death was ≥200 cells per cu.mm.

Univariate analysis found those patients whose baseline functional status was nonworking (p<0.001), those who were tobacco users before starting ART (p=0.019), those who were alcoholics before (p=0.003) and after (p=0.001) starting ART, those with OIs and tuberculosis before (p<0.001) and after starting ART (p<0.001), patients who were in WHO Clinical Stage 4 of HIV disease before initiation of ART (p<0.001), those who had ≤15 visits to treatment centre (p<0.001), those who took ART for ≤2 years (p<0.001), those whose adherence to ART was either average or poor (p=0.03), and patients from urban areas (p<0.001) were significantly associated with mortality status. There was no association between age (p=0.602), gender (p=0.537), marital status (p=0.287), educational status (p=0.598), occupation (p=0.06), history of smoking before starting ART (p=0.582), tobacco chewing after starting ART (p=0.903), smoking after starting ART (p=0.817), health status of the spouse (p=0.125), clinical staging of HIV at different intervals during the observation period after starting ART (p= ns), type of ART (p=0.54), age at starting ART (p=0.627), monthly income (p=0.588), BMI (p=0.624), and time since diagnosis to start of ART (p=0.133) with mortality status among patients in this study.

Mean CD4 count (cells per cu.mm) before the start of ART among patients who survived (n=241) was 402.5±235.3 in comparison to 266±206.1 among those who died (n=44). This difference in the mean CD4 counts was significant using unpaired t test, t=3.602, p<0.001.

There was no significant difference in the mean values of base line total leucocyte count (p=0.7) and base line haemoglobin levels (p=0.454) between patients who survived and died.

To calculate unadjusted hazard ratio, WHO clinical stage 1 of HIV was taken as reference value. Cox's regression was applied to the mortality data using variables significantly associated with it and the output is presented as Table 3.

A patient with any OI before the start of treatment was 2.251 times more likely to die in comparison to patient without any OIs. Similarly, patients with tuberculosis before starting treatment, unsatisfactory (poor/average) adherence to ART, and those with history of alcohol usage during treatment were at 2.22, 2.564 and 1.348 times, respectively, at greater risk of dying when compared with the rest. The confidence interval of these observation does not contain 1, indicating statistical significance (Table 3).

**Figure 1:**
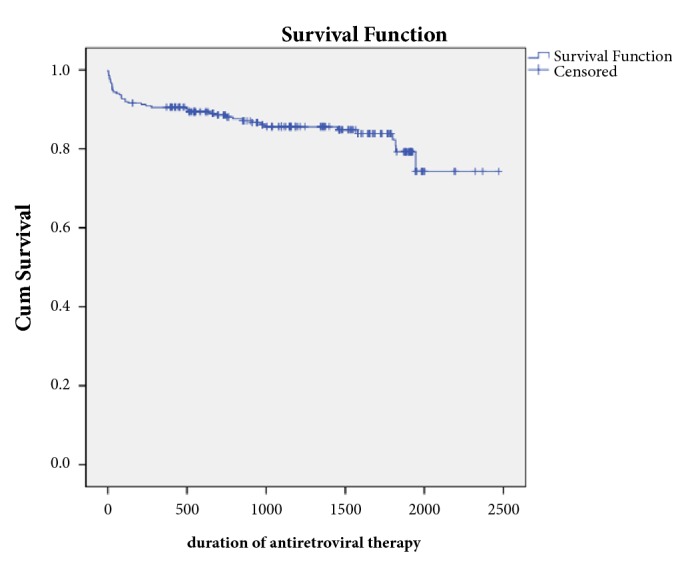
Kaplan-Meier survival curves illustrating cumulative survival probability of people living with HIV after initiation of ART (n=285).

The cumulative proportion surviving was plotted against the survival times. This resulted in a stepped survival curve presented as Figure 1. The mean survival time after initiation of ART was found to be 2084.0±55.3 days (95% CI is 1975.5-2192.5).

The mean survival time for patients after detection of HIV in this study was 5583.5±249.3 days (95% CI is 5094.8-6072.2).

**Figure 2:**
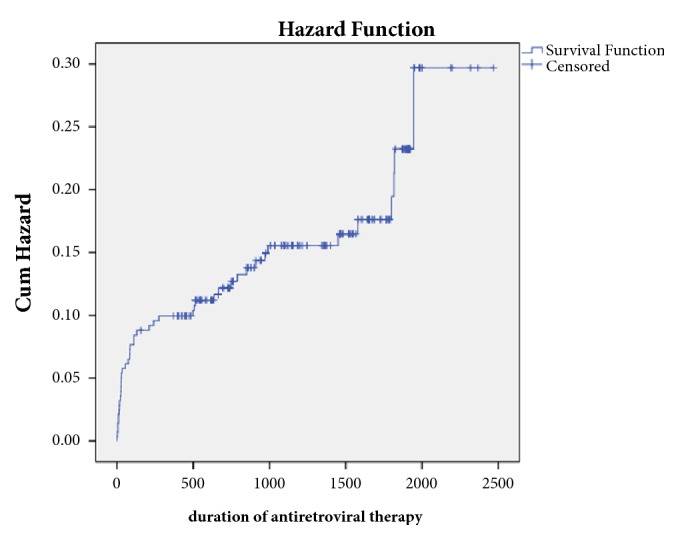
Cumulative hazard function of people living with HIV after starting ART (n=285).

The hazard function h(t) which is the conditional probability of dying at time t is shown in Figure 2.

## 4. Discussion

This study was done to identify the prognostic factors influencing mortality among a cohort of HIV infected adult patients on ART. It was observed that the presence of OIs and tuberculosis before and poor/average adherence to ART and alcohol usage after starting ART were independent prognostic factors of mortality among these patients.

The incidence density of death rate in this study was 3.12 per 100 PYO compared to other studies, where it was 1.75 [12], 2.03 [3], 3.4 [13], 3.8 [8], 5.1 [11], 7.6 [10], and 20.2 [14] PYO, respectively.

The proportion of patients who died in this study was 15.4% in comparison to two other studies done in Ethiopia, where it was reported as 9.4% [13] and 11.1% [11]. The proportion of patients who died was highest four or more years following diagnosis with HIV in the present study, as also observed in other studies [11, 15]. The proportion of deaths during this phase was 14.1% [11] and 21% [10, 15] in comparison to 5.5% observed in this study. The wide variation in these observations as reported in different studies could be due to varying time of enrolment into ART care.

This study observed that the mean survival time was 46.7 months, among patients with the most recent CD4 count before death, as ≥200 cells per cu.mm. This was more than 44.2 months reported in a study done in Hyderabad, India [10]. This infers that the survival probability of patients in the present settings was better than that reported elsewhere. This may be due to their better access to ART and good follow-up visits to treatment centres.

Majority of deaths in this study occurred within 3 months of starting ART, as also observed in previous studies [8, 16, 17]. However other studies reported majority of deaths during first 4 months [7], 5 months [15], 6 months [10, 18–20], and within 1 year [3, 12] of starting ART. Early mortality among patients on treatment may be due to undiagnosed OIs [21]. Other reasons cited for this could be, late diagnosis leading to delay in starting ART at a time when the disease has progressed to the later stages [5]. These observations emphasize the importance of early diagnosis and initiation of ART for all PLHIV's. This needs to be supported with careful monitoring of patients during the early phase of treatment.

In this study, good adherence to ART was reported in 93.7% patients in comparison to 27.4% [20], 94.2% [15], and 97.5% [8] reported in other studies. Medication adherence is very essential to utilize maximum benefit of ART. Nonadherence increases risk of drug resistance and early treatment failure. The side effects of treatment which were associated with unsatisfactory adherence among patients in the present study need timely management to ensure good compliance with treatment.

In a study done in Ethiopia, an 8 percentage gain in weight was observed in the first 6 months of treatment [11]. However, minimal change in median weight was noticed in the subsequent months. In comparison, in this study there was notable increase in BMI after 6 months of ART, indicating good response with continual treatment.

Progressive change in CD4 count after initiation of ART was noticed in this study. However the increase in median CD4 count level from baseline values over the first 6 months of ART was lesser compared to 76.2% reported in an Ethiopian study [11].

Multivariable analysis in the present study showed that the presence of OIs, including tuberculosis before starting ART, to be associated with greater risk of mortality. Therefore, early identification of OIs will be helpful in identifying patients who need more intense follow-up for treatment of specific OI.

Similarly, the presence of tuberculosis infection as an independent prognostic factor of mortality was reported by previous studies [8, 11, 12, 15]. It is a known fact that mycobacterium tuberculosis can cause tuberculosis in HIV patients at any stage of the disease, irrespective of the severity of immunosuppression. It also features among the leading cause of death in HIV infection which might be the reason for this observation.

Another important independent prognostic factor of mortality observed in this study was poor/average pattern of adherence to ART, which was also supported by other studies [15, 22]. Careful follow-up of patients with unsatisfactory adherence, timely management of side effects related to ART, and providing them drug counselling for compliance are crucial to improve survival of HIV patients on treatment.

Further, history of usage of alcohol after starting ART was also an important prognostic factor of mortality. This was probably because it causes poor adherence with treatment. Another Ethiopian study too observed that patients reporting substance usage, with significant independent risk of mortality [12]. Avoidance of alcohol during course of treatment by counselling constitutes additional supportive measures for improving survival of PLHIV.

Previous studies using Cox regression analysis identified male gender [14, 15, 19, 23, 24], single marital status [11, 15], bedridden functional status [3, 11–13, 23], advanced WHO clinical stage [3, 8, 11–15, 22–25], underweight [11, 13, 14, 19], low CD4 count [3, 11, 14, 19, 22, 23], severe anaemia [8, 11, 14, 19, 22, 25], and older age [13, 15, 19, 23] as independent risk factors of mortality among PLHIV which was not seen in this study. There was no hazard difference for low and high CD4 count in this study, as three-fourth of the patients had CD4 count ≥200 cells per cu.mm. Also no hazard difference between early and advanced stage disease in this study supports the importance of early initiation of ART therapy irrespective of the clinical stage of the disease.

The proportion of deaths was minimal 4 years after diagnosis of HIV in this study when compared with previous studies. Similarly, the survival probability of patients was better than that reported in other settings, probably due to acceptable level of adherence to treatment and good number of follow-up visits by the patients. The CD4 count and BMI were found to increase among them, as soon as 6 months of treatment. However, majority of deaths were reported within 3 months of starting ART. This could be due to delayed diagnosis of HIV and associated OIs. Added to this, side effects of ART need to be attended to during follow-up visits. Therefore intensive monitoring of HIV patients on ART with various factors identified in multivariable analysis such as presence of OIs before the start of treatment and counselling for the alcoholics and those with poor/average adherence to treatment (probably due to drug side effects) is a must for prolonging the survival of the HIV affected patients in this settings.

## 5. Conclusion

This study highlights the mortality statistics among PLHIV and the factors influencing it in an urban tertiary care setting of coastal India. It was observed that clinical and simple laboratory data available to health care providers can predict patients on treatment who are at higher risk of mortality. Screening efforts need to be intensified for early detection and management of OIs among HIV infected patients both before and after the start of ART. Other measures like good counselling and periodic monitoring would improve adherence and might also prevent alcohol consumption after treatment with ART. Monitoring and frequency of visits need to be more frequent in the first 3 months of starting ART, during which the proportion of deaths was found to be the most. These measures would help a long way in reducing mortality among PLHIV in the setting.

## 6. Limitation

This being a record based study has the limitation of availability of information, as stated in the records. There is also a possibility of deaths in this study which may have occurred due to causes not directly related to HIV/AIDS. The retrospective study design which limits analysis of the role of social and psychological factors and a single centric study which limits generalizability of information are other shortcomings of this research study.

## Figures and Tables

**Table 1:** Demographic and clinical characteristics of HIV patients.

Demographic characteristics	Number	Percentage

Age group (years)		

18-25	6	2.1

26-35	28	9.8

36-45	111	39.0

46-55	94	33.0

56-65	40	14.0

>65	6	2.1

Gender		

Male	176	61.8

Female	109	38.2

Marital status		

Unmarried	46	16.1

Married	177	62.1

Divorced	9	3.2

Widow/ widower	53	18.6

Educational status (n=278)		

Illiterate	25	9.0

Primary school	129	46.4

Secondary school	81	29.1

College and above	43	15.5

Occupation (n=269)		

Unemployed	87	32.3

Unskilled	54	20.1

Semi-skilled	70	26.0

Skilled	32	11.9

Semi-professional	7	2.6

Professional	19	7.1

Monthly income (n=239)	Median INR 5000 (IQR 3000- 8000)	

Health condition of the spouse (n=203)		

Healthy	102	50.2

Chronically ill	61	30.1

Died	40	19.7

Place		

Urban	243	85.3

Rural	42	14.7

Clinical characteristics	Number	Percentage

Type of ART		

First line	266	93.3

Second line	19	6.7

Mean age at diagnosis of HIV (years)	41.1±10.3	

Age at starting ART (years)		

≤20	4	1.4

21-30	20	7.0

31-40	91	31.9

41-50	112	39.3

51-60	51	17.9

>60	7	2.5

Time between diagnosis and initiation of ART (days)	Median 55 (IQR 7 - 910)	

Duration of taking ART (days)	Mean 1127.0±611.8	

Time between initiation of ART and death (days) (n=44)	Median 110 (IQR 25.2 - 780)	

Cotrimoxazole prophylaxis		

Yes	73	25.6

No	212	74.4

Isoniazid prophylaxis		

Yes	43	15.1

No	242	84.9

Adherence to ART		

Good	267	93.7

Average	14	4.9

Poor	4	1.4

Side effects of ART		

Present	35	12.3

Absent	250	87.7

Mean number of visits to ART centre	30.4±16.5	

Outcome of treatment		

Survived	241	84.6

Died	44	15.4

Total	285	100.0

**Table 2:** Distribution of various characteristics before and after start of antiretroviral therapy (ART) among patients.

Characteristics before starting ART			Characteristics after starting ART		

	Number	Percentage		Number	Percentage

Functional status (n=285)			Functional status (n=240)		

Ambulatory	51	17.9	Ambulatory	1	0.4

Bedridden	6	2.1	Bedridden	1	0.4

Working	228	80.0	Working	238	99.2

Pattern of substance usage (n=285)			Pattern of substance usage (n=285)		

Tobacco chewing	13	4.6	Tobacco chewing	14	4.9

Smoking	32	11.2	Smoking	15	5.3

Alcohol consumption	62	21.7	Alcohol consumption	22	7.7

Opportunistic infections present (n=285)			Opportunistic infections present (n=285)		

Extra pulmonary tuberculosis	34	11.9	Pulmonary tuberculosis	15	5.3

Pulmonary tuberculosis	28	9.8	Extra pulmonary tuberculosis	9	3.2

Candidiasis	5	1.7	Cytomegalovirus infection	2	0.7

Pneumocystis jiroveci pneumonia	4	1.4	Toxoplasmosis	2	0.7

Cryptococcal infections	2	0.7	Others*∗∗*	2	0.7

Toxoplasmosis	2	0.7			

Others*∗*	3	1.0			

BMI (Kg per sq.mtrs) of patients before starting ART (n=257)			BMI of patients after 60 months of starting ART (n=67)		

Under weight	87	33.9	Under weight	19	28.4

Normal weight	112	43.6	Normal weight	23	34.3

Over weight	25	9.7	Over weight	16	23.9

Obese	33	12.8	Obese	9	13.4

Clinical staging of HIV patients before starting ART (n=285)			Clinical staging of HIV patients after 60 months of starting ART (n=90)		

Stage 1	172	60.3	Stage 1	75	83.3

Stage 2	18	6.3	Stage 2	6	6.7

Stage 3	27	9.5	Stage 3	5	5.6

Stage 4	68	23.9	Stage 4	4	4.4

Mean CD4 count (cells/cu.mm) before starting ART (n=285)	381.4±235.9		Mean CD4 count (cells/cu.mm) after 60 months of starting ART (n=62)	596±216.7	

*∗*Cytomegalovirus infection 1, recurrent respiratory infections 1, and cellulitis 1.

*∗∗*Cryptococcal infections 1 and skin rashes 1.

**Table 3:** Multivariable Cox regression analysis of prognostic factors of mortality among HIV patients after starting ART (n=285).

Characteristics	Unadjusted HR	95% CI of unadjusted HR		P value	Adjusted HR	95% CI of Adjusted HR		P value

		Lower	Upper			Lower	Upper	

Presence of opportunistic infections before starting ART	3.504	1.691	7.26	<0.001	2.251	1.047	4.839	0.038

Presence of tuberculosis before starting ART	6.872	3.31	14.266	<0.001	2.22	1.074	4.592	0.031

Functional status at baseline	4.06	2.037	8.093	<0.001	0.644	0.442	0.936	0.021

WHO clinical staging of HIV at baseline	4.624	2.295	9.314	<0.001	1.266	0.965	1.66	0.089

Unsatisfactory adherence to treatment	3.013	1.067	8.511	0.03	2.564	1.001	6.571	0.05

History of alcohol usage after starting ART	4.51	1.795	11.332	0.001	1.348	1.02	1.781	0.036

## Data Availability

The data used to support the findings of this study are available from the corresponding author upon request.
